# Tuning the Energy Levels of Adamantane by Boron Substitution

**DOI:** 10.3390/molecules30091976

**Published:** 2025-04-29

**Authors:** Aminu H. Yusuf, Vladimir B. Golovko, Sarah L. Masters

**Affiliations:** School of Physical and Chemical Sciences, University of Canterbury, Private Bag 4800, Christchurch 8140, New Zealand; aminu.yusuf@pg.canterbury.ac.nz

**Keywords:** adamantane, bora-adamantane, DFT, CCSD(T), HOMO–LUMO, optical properties, electronic properties

## Abstract

Adamantane is known to have two different carbon environments, the C1-type (or bridgehead) and C2-type (or methylene bridge), serving as a foundation to explore the effects of boron substitution at these sites. Using DFT with B3LYP/6-31G(d), the structural, electronic, and optical properties of 37 boron-substituted isomers were investigated. The adamantane structure has rigid *T_d_* symmetry with an average *r*C-C of 153.7 pm, which progressively transforms to *C*_3v_ and *C*_1_ symmetry in heavily substituted isomers. Analysis of the neutral and ionic species reveals a critical transition from electron-donating to electron-accepting behaviour at tri-boron substitution, confirmed by both DFT and coupled cluster calculations (CCSD(T)/CC-pVDZ). C1 substitution narrows the HOMO–LUMO gap significantly, achieving a 56% reduction compared to 44.5% for C2 substitution in tetra-bora derivatives compared to adamantane. Optical properties [CAM-B3LYP/6-311G(d,p)] show systematic red shifting with increasing boron substitution, with absorption maxima moving from 146 nm in pristine adamantane to 423 nm (C1) and 277 nm (C2) in heavily boron-substituted derivatives (tetra-bora-adamantane). While C1 substitution leads to symmetry-forbidden transitions, C2 substitution maintains allowed transitions, offering more consistent optical behaviour. These findings provide important insight for the design of adamantane-based materials with tailored electronic and optical properties.

## 1. Introduction

**Figure 1:**
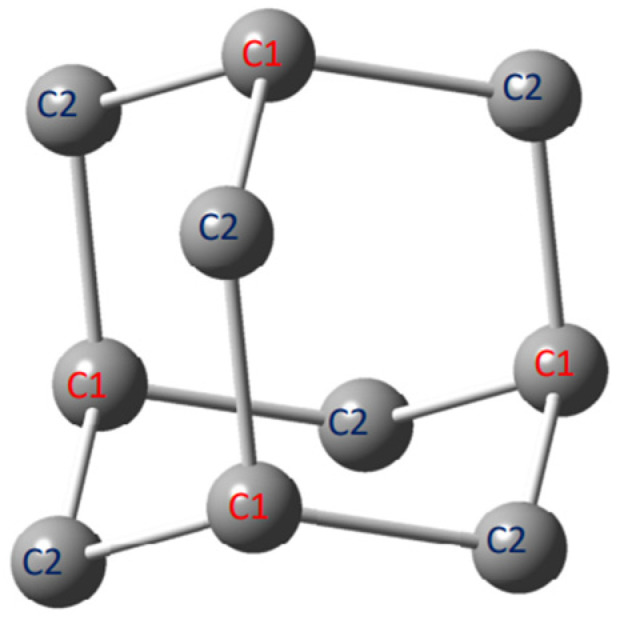
Pristine adamantane showing the two different types of substitution site (C1 and C2).

Adamantanes (C_10_H_16_) represent the simplest member of diamondoids, a class of cage-like polycyclic hydrocarbons with the general formula C_4n+6_H_4n+12_. The parent structure exhibits high symmetry (*T_d_*) with two distinct types of carbon positions comprising four tertiary bridgehead positions (C1-type) and six methylene bridges (C2-type; Figure 1) [1,2]. Natural sources of diamondoids include petroleum crude oils [3,4] and natural gas reservoirs [5], or they can be synthesized through chemical processes [6] such as Lewis acid catalysed hydrocarbon rearrangement [7].

Experimental and theoretical investigations reveal a large energy gap between the highest occupied molecular orbital (HOMO) and lowest unoccupied molecular orbital (LUMO) in these compounds [8,9,10]. However, through substitution and functionalization, the physical and chemical properties of these molecules can be tuned for applications in optoelectronics and other electronic devices [11,12,13]. This can be achieved through substituting either carbon or hydrogen atoms in the molecule with other atom(s) or functional groups. These changes can enhance the electronic and optical properties of diamondoids [14,15,16]. The unique physicochemical properties of these functionalized molecules have led to their investigation for nanotechnology applications such as N_2_ gas capture [17,18], energy storage devices [19,20], and medicine [21].

Therefore, substituting carbon atom(s) in the adamantane cage with electron rich or deficient elements such nitrogen or boron should enhance its electronic and optical properties [22,23]. Studies on BN-substituted adamantane by Wilson et al. demonstrated their remarkable stability and light absorption near the visible region, making them potential candidates for many optoelectronic applications [22]. Investigation into Group 14 elements has shown how the incorporation of silicon (Si_10_H_16_) and germanium (Ge_10_H_16_) modifies the adamantane structure [24]. These analogues exhibit larger exciton binding energies than pristine adamantane (C_10_H_16_), due to the delocalized LUMO state, which increases the shielding effects between the electrons and nucleus. The reduced optical gap in the heavier analogues results from quantum confinement effects that impact both the HOMO and LUMO states, whereas only the HOMO is affected in adamantane. Therefore, a pathway exists to tune properties in heavier analogues.

Functionalization by various groups such as alkali metals (Na, Li, and K) [25,26,27,28], chalcogens (S and O) [29], halogens [30,31] or other organic substituents such as hydroxyl [16], amines [32], thiols [16,33] and alkyls [34] can enable further electronic and optical modification of adamantane. Wu et al. demonstrated that single alkali metal substitution induces visible light absorption, enhanced first hyperpolarizability, and charge transfer effects, which are desirable for nonlinear optical applications [28]. Krongsuk et al. also confirmed changes in electronic and optical properties of adamantane by substituting hydrogen with up to four atoms of Li, Na or K. The substituted molecules appeared to have lower a HOMO–LUMO energy gap, increased electron affinity and enhanced dipole moments. These changes produce tunable visible region optical responses, with effects scaling according to substituent type and quantity [26]. Santo et al. investigated chalcogen functionalization in adamantane by studying the bond order effect on its optoelectronic properties. They added double-bonded CH_2_ radicals to the six methylene bridge carbons and replaced them with sulfur or oxygen at various positions [29]. Simultaneous substitution with sulfur and oxygen lowered the optical gap, with three sulfur substitutions achieving the lowest optical gap (1.47 eV), shifting the absorption spectrum to the visible region, beneficial for dyes and optoelectronic applications.

Research on carbon-based materials continue to reveal the diverse properties exhibited by carbon allotropes and their derivatives [35]. Boron substitution has emerged as a powerful approach to alter the electronic structure and optical properties of carbon-based materials such as graphene and nanotubes [36]. The work by Ekimov et al. on boron-doped diamond provides context for understanding how doping can modify the properties of *sp*^3^ carbon systems like adamantane [37]. Fyta and coworkers work on BN-based adamantane has also contributed to understanding of the effects of functionalization on structural, electronic and optical properties of these molecules [38]. However, the effect of boron substitution on the optical and electronic properties of adamantane has not been fully studied. Our work presented here uses density functional calculations to investigate the impact of selective boron substitution at different positions in the framework of adamantane. Specifically, we investigate how replacing CH groups with B atoms at bridgehead positions (C1-type) or CH_2_ groups with B-H at bridge positions (C2-type) modifies the fundamental properties of the adamantane cage.

## 2. Results and Discussion

### 2.1. Structure Modelling

Adamantane contains two different carbon environments. The first carbon type (C1-type or bridgehead) is bound to three other carbon atoms (C2-type) and one H atom, the second carbon type (C2-type or methylene bridge) is bound to two other carbon atoms (C1-type) and two H atoms. Gaussview [39] was used to systematically construct 37 different boron-substituted adamantane molecules by substitution of either the C1-type position in the ring by a boron atom (B1-type), or the C2-type position by B-H (B2-type). Various isomers are possible for each substitution combination. Table 1 presents the lowest-energy configurations for both C1 and C2 substitution patterns, with the complete isomer set detailed in Table S1 of the Supplementary Materials.

### 2.2. Structural Properties

Structural analysis of pristine adamantane reveals average bond lengths of *r*C-H = 108.8 pm and *r*C-C = 153.7 pm. The *r*C-C value aligns well with previous theoretical calculations (153.8 pm) [40] and experimental crystallographic measurements (153 pm) [41,42]. Progressive boron substitution at C1-type and C2-type positions induces systematic changes in molecular geometry. The remaining *r*C-C bonds exhibit sequential elongation, reaching approximately 160 pm in the hexa-bora adamantane isomers. *r*C-B is also noticeably elongated with increasing boron substitution. Angular distortion is also evident, with ∠C-C-C expanding from 109.7° to 117.6° in the heavily substituted hexa-bora adamantane.

Boron substitution in adamantane distorts the symmetry, and this can be explained by Wade’s rule [43], which predicts the shape of the compounds formed by main group elements. Pristine adamantane (C_10_H_16_) is thought to have carborane-like structure, having a closo-cluster with 10 vertices and 32 skeletal electrons, adhering to Wade’s (n + 1)^2^ rule, adopting a highly symmetric structure (*T_d_* symmetry). Substituting a carbon atom with a boron atom reduces the number of skeletal electrons, lowering the symmetry to *C*_3V_ and eventually to *C*_1_ with more boron being substituted, due to inward relaxation of the of neighbouring atoms caused by smaller atomic radius of boron. This aligns with the theoretical studies on boron aggregation in diamond crystals [44], where introduction of boron creates electron-deficient centres and causes inward relaxation of the neighbouring atoms, leading to simultaneous breaking of the symmetry.

Our DFT calculations yielded a total electronic energy of −10632 eV for pristine adamantane, similar in magnitude to the −10635.4 eV reported by Wilson et al. [22] using plane wave DFT. While these absolute values cannot be directly compared across computational methods, their similar magnitude provides general validation of our methodology. Boron substitution at various substitution sites resulted in a progressive increase in electronic energy with mono and hexa-bora substituted isomers exhibiting lowest and highest energies of −10254 eV and −8370 eV, respectively. Substitution at the C1 and C2 positions produced isomers of nearly identical energy levels, with the C1 isomers being slightly more stable by 0.01–0.05 eV. This marginal difference between C1 and C2 isomers indicates that both positions are energetically favourable for boron substitution. This trend of increasing total energy with higher boron substitution is reported in other theoretical studies on boron-doped carbon material [45,46], reflecting increased structural strain and electronic reorganization in the rigid-cage framework. The lowest-energy isomer from each substitution pattern (C1 and C2) were selected for subsequent detailed analysis of electronic and optical properties. A comprehensive examination of the neutral and ionic species energetics, which provides deeper insight into the electronic characteristics and stability of these systems, is presented in the following section. Detailed structural parameters and optimized geometries are provided in Supplementary Materials (Figure S1 and Table S2).

### 2.3. Electronic Properties

#### 2.3.1. Analysis of Neutral, Anionic and Cationic Energies

The electronic structure properties for pristine adamantane and its boron-substituted derivatives reveal significant trends in stability and reactivity. This work focused on analysis of the ground-state energies of the neutral, cationic, and anionic species, to gain insights into their electronic behaviours without relying on direct calculations of ionization energies (IE) and electron affinities (EA), which can be problematic in DFT calculations [47]. While IE and EA are commonly used to describe electronic properties, direct calculation of these values using density functional theory (DFT) can be challenging due to limitations in accurately accounting for the energies of free electrons, exchange energy and correlation effects, particularly in systems with unpaired number of electrons [48]. Additionally, there exists ambiguity regarding the sign of EA, as various studies report positive and negative EA values for adamantane [49]. Thus, the relative energies of neutral, cationic, and anionic species were analysed and the two key energy differences that provide electronic behaviour and reactivity of these molecules was determined using Equations (1) and (2).

(1) $$∆E_{1}=E_{+}-E_{0}$$

(2) $$∆E_{2}=E_{0}-E_{-}$$

$E_{0}$, $E_{-}$ and $E_{+}$ are the total electronic energy of the optimized molecule in neutral, anionic, and cationic forms, respectively, while $∆E_{1}$ represents the energy difference between the neutral and positively charged species (reflecting the energy required to remove an electron) and $∆E_{2}$ is the energy difference between the neutral and negatively charged species (reflecting the energy released when adding an electron). These energy differences are analogous to the IE and EA, respectively, but differ experimentally as it does not account for instantaneous removal/addition of electrons without geometric relaxation. Our ∆E includes full geometric relaxation of the charged species.

Table 2 shows the calculated energies of neutral and ionic species, along with their corresponding energy differences, for pristine adamantane and its boron-substituted derivatives. The data shown represent the lowest-energy isomers for both C1 and C2 substitution patterns [complete data for all isomers are available in the Supplementary Materials (Table S3)].

The total electronic energy of the neutral species increases progressively with boron substitution for both C1 and C2 substitutions. The energy of the neutral molecules, *E₀* increases from −10632.18 eV in pristine adamantane to −8370.50 eV in 2,4,6,8,9,10-hexa-bora adamantane (C2 substitution). This trend suggests a progressive destabilization of the molecular framework with increasing boron content. The energy of the cationic species also increases with boron substitution, but at a slightly different rate compared to the neutral species. For C2 substitution, *E*_+_ increases from −10623.09 eV in pristine adamantane to −8361.30 eV in hexa-bora adamantane. The energy difference between neutral and cationic species ($∆E_{1}$) maintains consistently positive values throughout the series, ranging from 8.56 to 9.05 eV for C1 substitution and 8.54 to 9.20 eV for C2 substitution. This positive $∆E_{1}$ indicates that electron removal remains energetically unfavourable across all substitution levels, though the increasing values at higher substitution levels suggest a growing resistance to electron loss.

Stability of the anions is characterized by three distinct regions in the $∆E_{2}$ values, demonstrating a remarkable transition from electron-donating to electron-accepting character. The first region, encompassing pristine and mono-substituted species, shows strong resistance to electron addition ($∆E_{2}<-1.0\;eV$), evidenced by a substantial negative $∆E_{2}$ value of −3.36 eV in pristine adamantane. This indicates significant instability of the anionic species relative to the neutral molecule ($E_{-}>E_{0}$), consistent with known electronic properties of adamantane [24,33,50]. The second region, observed in di-substituted species, represents a metastable state $(-1.0\;eV<∆E_{2}<0\;eV$) where $∆E_{2}$ values further decrease in magnitude to −0.35 eV and −0.25 eV for C1 and C2 substitutions, respectively. The third region, beginning with tri-substituted species (both C1 1,3,5-tri-bora- and C2 2,4,6-tribora-adamantane), demonstrates favourable electron acceptance ($∆E_{2}>0\;eV$), marking a complete transformation in electronic behaviour.

To validate the DFT results, coupled cluster calculations on the lowest-energy isomers for both C1 and C2 substitution were undertaken at CCSD(T)/CC-pVDZ level [51,52]. These calculations demonstrate that DFT yielded relative energies consistent with coupled cluster values for both C1 and C2 substitution patterns. However, the DFT absolute energies overestimate stability (lower energies) compared to coupled cluster, with mean absolute error of 27.78 eV reflecting the known limitation of B3LYP for calculating absolute energies [53]. This deviation in absolute energy is highlighted for the key substitution in Table S4. Examining the energy differences, both methods yield results with similar magnitudes, the parent adamantane showing CCSD(T) values of 9.83 eV and −3.18 eV compared with DFT values of 9.10 eV, −3.36 eV for $∆E_{1}$ and $∆E_{2}$ respectively. This quantitative difference is attributed to a higher accuracy associated with CCSD(T) calculations, which explicitly account for electron correlation effects [54]. However, the results prove the reliability of B3LYP in predicting these energetic properties in molecular systems with less computational cost.

Additionally, for the critical transition points, the couple cluster calculations revealed neutral species energies of −9852.07 eV and −9475.77 eV, respectively, and anionic species energies of −9851.60 eV and −9476.20 eV for 1,3-di-bora and 1,3,5-tri-bora adamantane, respectively. This gives coupled cluster calculation-based values of $∆E_{2}$ = −0.47 eV for 1,3-di-bora and $∆E_{2}$ = 0.43 eV for 1,3,5-tri-bora adamantane for C1 substitution. The same trend is observed for C2 substitution as shown in Table 3 below.

While the energies show method-dependent variations, both computational approaches capture the fundamental transition in electronic behaviour from electron-donating to electron-accepting at the tri-substituted adamantane. This consistent prediction across different computational methods provides strong evidence for the predicted electronic transition, suggesting it to be an intrinsic property of the system rather than a method-dependent property.

#### 2.3.2. Analysis of HOMO and LUMO Orbitals

**Figure 2:**
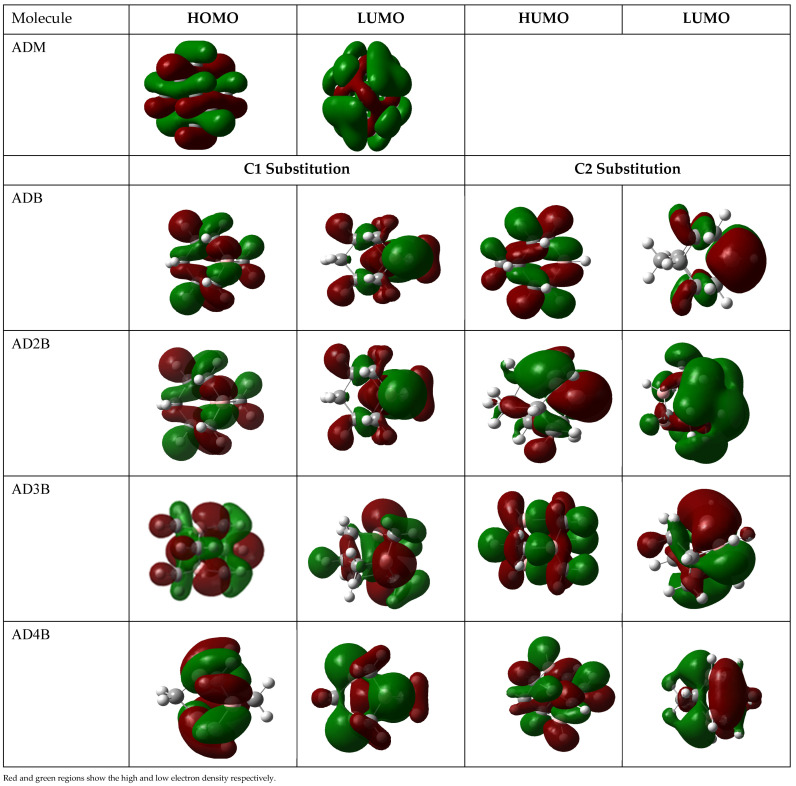
Ground-state frontier orbitals for the boron-substituted adamantane series using B3LYP/6-31G(d). (ADM = pristine adamantane, ADB = mono-bora-adamantane, AD2B = di-bora-adamantane etc.) Iso-surface value (0.02 e/Å^3^). Red and green regions show the high and low electron density, respectively.

The frontier molecular orbitals is used to examine the reactivity of the molecules by analysing nature of the HOMO and LUMO orbitals [55], as depicted in Figure 2. It is evident that the HOMO and LUMO orbitals of pristine adamantane are symmetrically distributed through the entire molecule suggesting delocalized electron density across the molecule consistent with its high symmetry. Upon substitution with boron, the charge distribution of the HOMO is concentrated to the interstitial region between C-B bonds while the LUMOs are localized over the substituted boron region with partial density on the C1-type and C2-type positions. This aligns with theoretical investigations using plane-wave DFT on bora-adamantane and tetra-bora-adamantane where boron is substituted at the C1 carbon positions [40]. There is a systematic alteration of the electronic structure, evident from the progressive changes in orbital sizes and shapes with increasing substitution. Notably, the HOMO of tetra-bora-adamantane substituted at C1 is highly localized on one side of the molecule due to the reduction in symmetry. Additionally, electron density on the LUMO increases, particularly in di-bora- and tetra-bora-adamantane substituted at the C2 site, indicating an enhance electron-accepting capability induced by boron substitution. These trends are likely to reduce the HOMO–LUMO gap leading to interesting electronic and optical properties.

**Figure 3:**
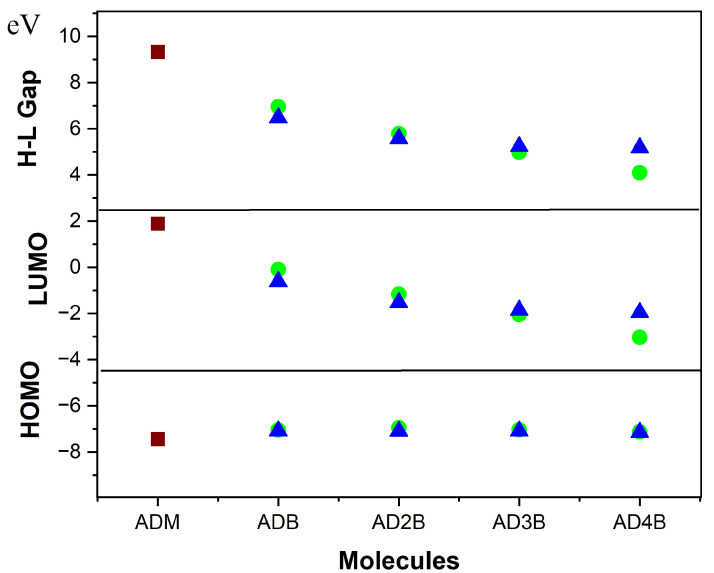
Effect of boron substitution on the orbital energies and energy gaps for the lowest-energy isomers of the boron-substituted adamantane series. ADM = pristine adamantane, ADB = mono-bora-adamantane, AD2B = di-bora-adamantane, etc. (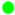
—C1-type substitution, 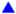
—C2-type substitution, and 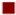
—pristine adamantane).

Unlike nitrogen substitution, which primarily affects HOMO energies through its lone pair [32], our boron-substituted adamantanes uniquely modify both HOMO and LUMO levels because of boron substitution at both the C1 and C2 sites as shown in Figure 3 [Orbital energies for all isomers are given in the Supplementary Materials (Table S5)]. Specifically, the HOMO energies increase from −7.44 eV in pristine adamantane to approximately −7.10 eV in the 2-bora, 2,4 di-bora and 2,4,6 tri-bora adamantane derivatives. before decreasing to −7.71 eV in the hexa-bora derivative for C2 type. A similar trend is observed for the C1 substitution type, although the HOMO energies are slightly higher in energy when compared to C2 substitution site with 1,3,5,7 tetra-bora adamantane having the lowest energy (−7.13 eV), followed by 1 bora and 1,3,5 tri-bora with −7.03 eV and 1,3 di-bora adamantane with the highest energy value (−6.95 eV). This decrease stabilizes the HOMO orbital and is linked to the formation of delocalized orbitals involving multiple boron centres as observed in a previous study [38]. The LUMO energy decreases with boron atom substitution, changing from a positive value (1.88 eV) in adamantane to a negative value (−0.09 and −0.62 eV) in mono-bora substituted adamantane at the C1 and C2 sites, respectively. C1 substitution leads to lower LUMO energies, suggesting enhanced electron-accepting properties compared to C2 type substitution. Additionally, a subsequent increase in the LUMO energies is observed in heavily substituted penta- and hexa-bora-adamantane. This trend can again be explained by Wade’s rules [43] with the electron deficiency introduced by boron substitution leading to deviation from the stable closo-structure of pristine adamantane. However, the penta- and hexa-bora-adamantane with higher boron content can still form stable delocalized bonding orbitals, resulting in increased LUMO energy. The HOMO–LUMO gap is reduced significantly by both substitution patterns, with C1 substitution leading to a more pronounced HOMO–LUMO gap reduction of 56% as compared to 44.5% with C2 substitution in tetra-bora-adamantane.

#### 2.3.3. Density of States Analysis

**Figure 4:**
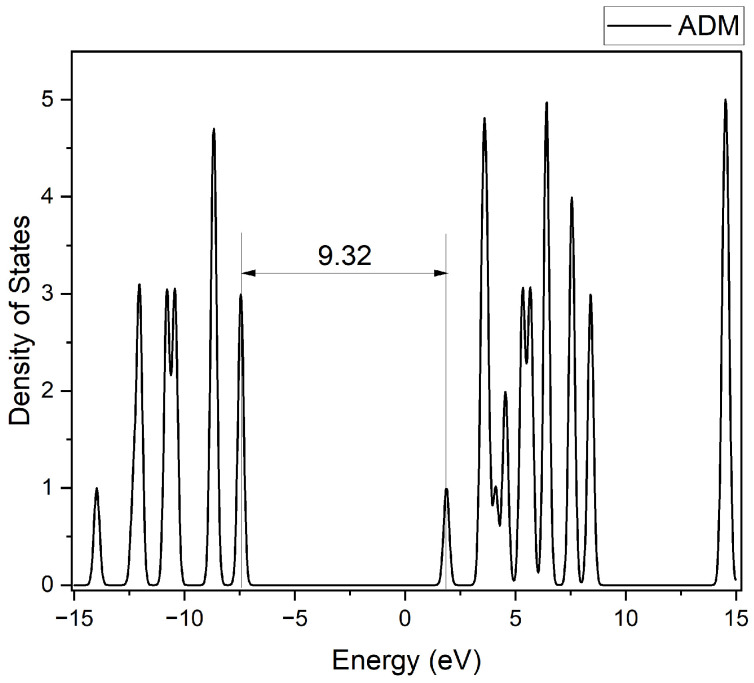
Density of states spectrum of pristine adamantane (ADM).

**Figure 5:**
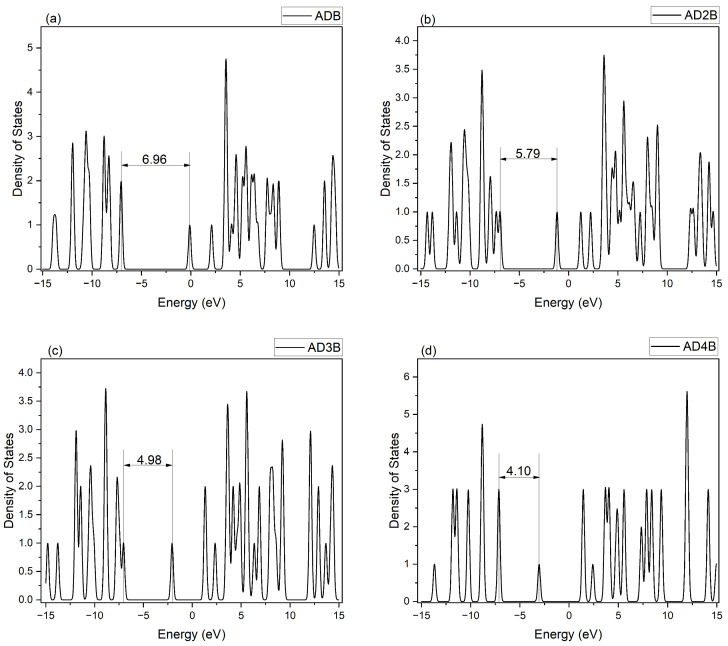
Density of States spectra for C1 substitution molecules: (**a**) ADB = mono-bora-adamantane, (**b**) AD2B = di-bora-adamantane, (**c**) AD3B = tri-bora-adamantane, and (**d**) AD4B = tetra-bora-adamantane.

**Figure 6:**
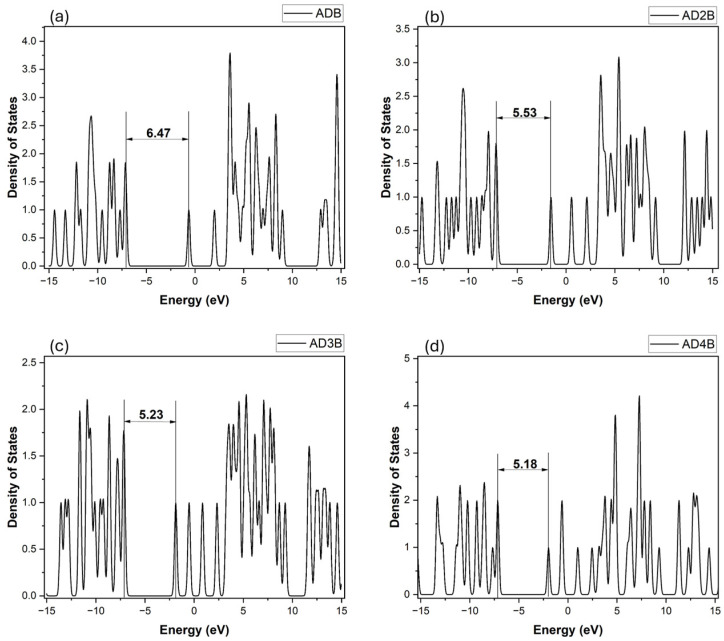
Density of States spectra for C2 substitution molecules: (**a**) ADB = mono-bora-adamantane, (**b**) AD2B = di-bora-adamantane, (**c**) AD3B = tri-bora-adamantane, and (**d**) AD4B = tetra-bora-adamantane.

The electronic density of states (DOS) for occupied and unoccupied states were generated using the GaussSum software (Version 3.0) [56]. The DOS plots for pristine adamantane and the lowest-energy B-substituted adamantane isomer for each substitution group (C1-type and C2-type) are presented in Figure 4, Figure 5 and Figure 6. As expected, pristine adamantane shows a wide energy gap of 9.33 eV between the HOMO and LUMO. In contrast, the substituted molecules reveal a trend of decreasing band gap with increasing boron substitution at both substitution sites. Both substitution patterns show increased complexity in the valence and conduction bands with increased boron content. The C2 substitution generally results in a smaller alteration of the electronic properties, maintaining larger band gaps and more symmetric DOS distributions around the Fermi level (0 eV). Conversely, C1 substitution leads to larger changes, characterized by narrower band gaps and asymmetric DOS distribution, evident in the tri and tetra-substituted structures. Additionally, there is the appearance of new states in the lower conduction band region as boron content increases, potentially introducing new electronic transition pathways. This observation correlates with the enhanced electron-accepting properties indicated by the relative energies of neutral and anionic species in heavily boron-substituted adamantanes.

### 2.4. Optical Properties and Transition Character

TD-DFT calculations using CAM-B3LYP/6-311G(d,p) were used to study the optical properties of adamantane and B-substituted adamantanes. The CAM-B3LYP functional incorporates range-separated exchange-correlation, making it more suitable for systems where electronic excitations may involve substantial charge redistribution [57]. Additionally, the 6-311G(d,p) basis set employed with CAM-B3LYP provides a more complete description of the orbital space. Table 4 shows the optical properties at lowest and most intense transitions for lower-energy isomers substituted at C1 and C2. Full data for all the molecules are shown in the Supplementary Materials (Table S6) and the natural transition orbitals (data from the TD-DFT calculations) is presented in Figure S2.

The pristine adamantane shows absorption at the region of 147 nm (8.44 eV) with weak transitions occurring between HOMO→LUMO and H-2→LUMO for both the primary and secondary transition, respectively. This is consistent with previous reports (146 nm) [29,32]. Boron substitution in adamantane significantly alters its optical properties, with C1 and C2 substitutions showing distinct trends.

For C1 substitution, a red-shifted absorption wavelength (423 nm) is observed particularly for 1,3,5,7-tetra-bora-adamantane, corresponding to a forbidden electronic transition from HOMO-1→LUMO. However, a low-intensity higher-energy transition (HOMO-5→LUMO) with oscillator strength of 0.0006 is also observed in the ultraviolet region around 238 nm. C2 substitution results in transitions that remain in the ultraviolet region, 2,4,6,8,9-penta-bora adamantane showing the highest absorption at 279 nm for the for the HOMO to LUMO transition and 206 nm for the higher-energy HOMO-4 to LUMO transition. The key intense transitions include 1-bora-adamantane at 153 nm (8.10 eV, oscillator strength 0.0952) and 2-bora-adamantane at 127 nm (9.76 eV, oscillator strength 0.1442) for both C1 and C2 substitutions.

The transition character evolution reveals systematic changes with increasing boron content. In unsubstituted adamantane, the lowest-energy transition exhibits a pure character involving symmetric HOMO→LUMO transitions with equal contributions from alpha and beta spin channels, consistent with the high molecular symmetry of adamantane. As the boron content increases, transitions begin to involve lower-energy occupied orbitals (H-1, H-2, H-4, H-6) and occasionally higher-energy unoccupied orbitals (L+1, L+2), particularly in C2 substitution. Most transitions maintain approximately equal contribution from alpha and beta spin channels, indicating that the electronic excitations preserve spin symmetry despite the structural modifications. Several transitions in boron-substituted derivatives show mixed character, involving contributions from multiple orbital pairs. For example, the transition at 174 nm in 1,3-dibora-adamantane shows mixed character with contributions from H-6(A)→LUMO(A) (37%) and H-3(A)→LUMO(A) (10%), indicating that the excitation involves electron density reorganization from multiple occupied orbitals. More complex mixtures appear in higher substituted derivatives, particularly in the C2 series, where transitions like that in 2,4,6,8,9-pentabora-adamantane (201 nm) involve four different orbital pair contributions [H-4(A)→LUMO(A) (16%), H-3(A)→L+1(A) (22%)].

There is limited experimental and theoretical data available on the optical properties of boron-substituted adamantanes. However, previous theoretical studies on electronic properties of BN substituted adamantane have suggested that functionalization at C2-type would give desirable properties with consequently stiffer nanostructure [40,58]. Our results show that substitution at both the C1-type and C2-type carbon can have a significant effect on the optical absorption of the boron-substituted adamantane. While boron substitution induces a substantial red shift (184–423 nm), particularly for tetra-substituted variants, other substituents show distinctive effects. For example, nitrogen-substituted adamantanes typically exhibit a blue shift, absorbing in the deep UV region (130–140 nm) [22,32]. Oxygen substitution introduces a moderate red shift (180–220 nm) and is characterized by n→π* transitions due to the presence of non-bonding orbitals [29]. Silicon-substituted derivatives show moderate red shifts (180–250 nm, 5.0–6.9 eV), but the effect is less pronounced than with boron [24,32], while halogen substitutions (F, Cl, Br) induce only slight red shifts (140–200 nm) typically showing a transitions from halogen-centered orbitals to cage σ* orbitals [31], a mechanism distinct from the deeper-orbital involvement seen in boron substitution.

These changes are attributed to the electronic perturbations induced by boron substitution, which affect the energy levels and transition probabilities. These findings provide valuable insights for the rational design of adamantane-based materials with tailored optical characteristics for potential applications in optoelectronic devices and photocatalysis.

### 2.5. Electrostatic Potential Surface

**Figure 7:**
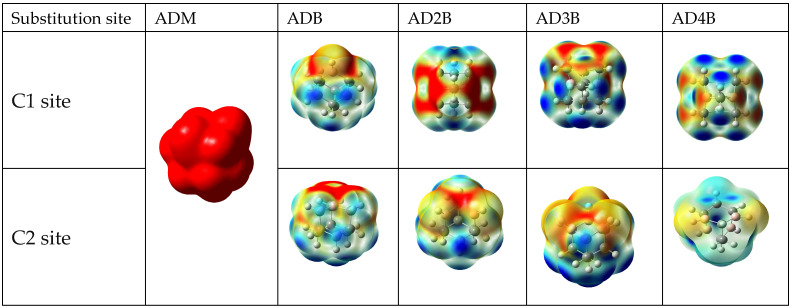
Electrostatic potential mapped surface for adamantane and the lowest-energy isomers of the boron-substituted adamantane series. ADM = pristine adamantane, ADB = mono-bora-adamantane, AD2B = di-bora-adamantane, etc.

Electrostatic potential (ESP) maps provide valuable insights into the charge distribution and reactivity of molecules and are widely used to predict nucleophilic and electrophilic reaction sites within molecules, as well molecular size and shape [24]. Figure 7 compares the ESP maps of pristine adamantane and its boron-substituted derivative, demonstrating systematic evolution of electronic structure with increasing boron content. The ESP surfaces are plotted with a color scale ranging from −0.02 (red) to +0.02 (blue) a.u., with intermediate values represented by yellow and green regions.

Pristine adamantane exhibits a uniform red coloration, indicating a homogeneous ESP across the molecule, consistent to its known non-polar nature and chemical inertness. In contrast, boron-substituted adamantanes show a multi-colored surface, revealing a heterogeneous electrostatic potential distribution. The red regions maintain high electron density (negative potential), while blue regions indicate areas of low electron density (positive potential), particularly around the boron substitution sites. The intermediate yellow and green regions represent gradual transitions in the ESP, with yellow indicating moderately negative regions and green showing near-neutral areas. This color spectrum becomes more pronounced with increasing boron content, demonstrating progressive electronic reorganization. A significant transition occurs at tri-substitution, with both C1 and C2 patterns showing a notable increase in blue regions, indicating increased electron-deficient character, with distinct distributions of yellow–green intermediate regions. This transition correlates with our observed changes in neutral and ionic species energetics, particularly the shift toward electron-accepting behaviour. The C1 tetra-substituted species show the most intense blue regions, with complex patterns of yellow and green transitional areas, demonstrating the cumulative effect of boron substitution on electronic structure. The differentiation between C1 and C2 substitution effects are visible in their distinct patterns of potential gradients and intermediate regions, this provides guidance on how substitution position influences electronic structure modification.

## 3. Computational Methods

All calculations were performed using the Gaussian 16 computational software package (Version C.01) [58]. Electronic structure calculations employed density functional theory (DFT) [59] with the B3LYP [60] functional and 6-31G(d) Gaussian-type double zeta basis set, a theoretical framework selected for its optimal balance between accuracy and computational efficiency in organic molecules [61].

The ground-state geometries were obtained and verified by frequency calculations for all molecules. Analysis of the vibrational frequencies confirmed convergence of all the calculations to minima on the potential energy surfaces. The ground-state energies of the neutral, cationic and anionic species were calculated for each molecule to evaluate the stability and reactivity of the substituted molecules. This approach was chosen over direct calculation of ionization energies and electron affinities to avoid known limitations of DFT in accurately representing isolated electrons [47]. The density of states (DOS) and the energy gap between highest occupied molecular orbital (HOMO) and lowest unoccupied molecular orbital (LUMO) were also analysed. Time-dependent density functional theory (TD-DFT) [62] with the CAM-B3LYP functional and 6-311G(d,p) was used to obtain optical properties such as absorption wavelength, excitation energy, electronic transition, and oscillator strength [63,64]. The UV–visible spectra were obtained and analysed, the nature of the frontier molecular orbitals and electrostatic potentials (ESP) was visualized using GaussView (Version 6). [39].

While DFT methods provide reasonable results for structural and energetic properties, they have known limitations in accurately describing electron correlation effects and dispersion interactions in excited states. To provide more accurate treatment of electron correlation and validate our DFT results, additional couple cluster calculations at CCSD(T)/CC-pVDZ level were conducted for the lowest-energy isomers [51,52]. Tables S7 and S8 in the Supplementary Materials detail some parameters calculated with larger basis sets for selected isomers.

## 4. Potential Applications and Future Prospects

The systematic modification of electronic and optical properties through controlled boron substitution in adamantane suggests several promising applications. The transition from electron-donating to electron-accepting character, coupled with tunable optical absorption, opens possibilities for application in optoelectronics devices (UV–visible detection, light-emitting devices, photodetectors), molecular electronics (charge transport materials, electronic switches), and energy applications (light harvesting, electron transport layers in solar cells). The well-defined structure-property relationships established in this work provide design principles for rational modification of molecular electronic properties and optical absorption characteristics, potentially guiding the development of other boron-modified carbon frameworks for specific applications.

While these computational predictions suggest potential of these molecules in various applications, experimental validation is necessary through comprehensive characterization including X-ray crystallography and NMR spectroscopy for structural verification, photoelectron spectroscopy, UV–visible spectroscopy and fluorescence spectroscopy for optical characteristics, and thermal analysis for stability assessment.

## 5. Conclusions

Using DFT and coupled cluster methods, the influence of increasing boron substitution on the structural, electronic and optical properties of adamantane was systematically studied. Optimized structures revealed that bonding properties vary with the number and position of B-substitutions. Predicted DFT total electronic energies indicated higher stability of neutral species compared to cations in all cases, while anions became more stable than the neutral species beyond tri-boron substitution. Coupled cluster calculations confirmed the shift in relative stability between di- and tri-substituted species for both C1 and C2 patterns.

Systematic boron substitution progressively destabilized the molecular framework while significantly enhancing electron-accepting capabilities at tri-substitution. Differences between C1 and C2 substitution emphasize the role of position in modulating electronic properties. TD-DFT optical studies showed that boron substitution altered absorption wavelengths and excitation energies, with most excitations corresponding to HOMO-1→LUMO and HOMO→LUMO transitions for C1 and C2 substitution, respectively. Both substitutions offer a shift in the absorption spectrum compared to parent adamantane, with broader wavelength found at 1,3,5,7-tetra-bora adamantane (423 nm), although indicating a forbidden transition.

These findings underscore the critical impact of the degree of substitution on the electronic properties of boron-substituted adamantane, with a secondary but notable influence of substitution position.

## Figures and Tables

**Table 1:** Lowest-energy isomers for each combination of boron-substituted adamantane showing structural configuration for two substitution position (C1-type or C2-type).

#	# Boron	Molecular Formula	Naming	Configuration of Different Atom Types ^a^
1	None	C_10_H_16_	Adamantane	(C1)_4_(C2)_6_
2	B	C_9_H_15_B	1-bora-adamantane	(C1)_3_(B1)_1_(C2)_6_
3		C_9_H_15_B	2-bora-adamantane	(C1)_4_(C2)_5_(B2)_1_
4	2B	C_8_H_14_B_2_	1,3-di-bora-adamantane	(C1)_2_(B1)_2_(C2)_6_
5		C_8_H_14_B_2_	2,4-di-bora-adamantane	(C1)_4_(C2)_4_(B2)_2_
6	3B	C_7_H_13_B_3_	1,3,5-tri-bora-adamantane	(C1)_1_(B1)_3_(C2)_6_
7		C_7_H_13_B_3_	2,4,6-tri-bora-adamantane	(C1)_4_(C2)_3_(B2)_3_
8	4B	C_6_H_12_B_4_	1,3,5,7-tetra-bora-adamantane	(B1)_4_(C2)_6_
9		C_6_H_12_B_4_	2,4,6,8-tetra-bora-adamantane	(C1)_4_(C2)_2_(B2)_4_
10	5B	C_5_H_11_B_5_	1,2,3,5,7-penta-bora-adamantane	(B1)_4_(C2)_5_(B2)_1_
11		C_5_H_11_B_5_	2,4,6,8,9-penta-bora-adamantne	(C1)_4_(C2)_1_(B2)_5_
12	6B	C_4_H_10_B_6_	1,2,3,5,6,7-hexa-bora-adamantane	(B1)_4_(C2)_4_(B2)_2_
13		C_4_H_10_B_6_	2,4,6,8,9,10-hexa-bora-adamantane	(C1)_4_(B2)_6_

^a^ Configuration indicates how many C1-type and C2-type carbons have been replaced with B1-type or B2-type. Hydrogen atoms have been omitted for clarity in the coding.

**Table 2:** Electronic energies and energy differences (eV) for the boron-substituted adamantane series calculated at B3LYP/6-31G(d) level.

Molecule	*E*_0_	*E*_+_	*E*_−_	Δ*E*_1_	Δ*E*_2_
Adamantane	−10,632.18	−10,623.09	−10,628.82	9.10	−3.36
C1 Substitution					
1-bora	−10,254.77	−10,246.21	−10,253.60	8.56	−1.17
1,3-di-bora	−9877.73	−9869.16	−9877.38	8.57	−0.35
1,3,5-tri-bora	−9500.93	−9491.88	−9501.44	9.05	0.51
1,3,5,7-tetra-bora	−9124.18	−9115.40	−9125.45	8.78	1.27
C2 Substitution					
2-bora	−10,254.56	−10,246.02	−10,253.45	8.54	−1.11
2,4-dibora	−9877.23	−9868.22	−9876.98	9.02	−0.25
2,4,6-tribora	−9499.90	−9491.19	−9500.50	8.71	0.60
2,4,6,8-tetrabora	−9122.80	−9114.07	−9123.36	8.73	0.56
2,4,6,8,9-pentabora	−8746.72	−8737.79	−8747.82	8.93	0.14
2,4,6,8,9,10-hexabora	−8370.50	−8361.30	−8371.43	9.20	0.93

**Table 3:** Electronic energies and energy differences (eV) for the boron-substituted adamantane series calculated at CCSD(T)/CC-pVDZ level.

Molecule	*E*_0_	*E*_+_	*E*_−_	Δ*E*_1_	Δ*E*_2_
Adamantane	−10,601.81	−10,591.98	−10,598.63	9.83	−3.18
C1 Substitution					
1-bora	−10,228.62	−10,219.14	−10,277.10	9.48	−1.52
1,3-di-bora	−9852.07	−9842.73	−9851.60	9.33	−0.47
1,3,5-tri-bora	−9475.77	−9466.36	−9476.20	9.41	0.43
1,3,5,7-tetra-bora	−9099.50	−9089.97	−9100.97	9.52	1.47
C2 Substitution					
2-bora	−10,228.36	−10,218.85	−10,227.31	9.51	−1.05
2,4-dibora	−9848.10	−9838.64	−9847.97	9.46	−0.13
2,4,6-tribora	−9471.33	−9461.97	−9471.58	9.36	0.25
2,4,6,8-tetrabora	−9094.81	9085.36	−9095.11	9.45	0.29
2,4,6,8,9-pentabora	−8719.38	−8709.72	−8719.66	9.66	0.27
2,4,6,8,9,10-hexabora	−8343.50	−8333.85	−8343.43	9.65	0.30

**Table 4:** Optical properties of adamantane and the lowest-energy isomer of various boron-substituted adamantanes showing lowest and most intense transitions [CAM-B3LYP/6-311G(d,p)].

Molecules	Absorption Wavelength (nm)	Excitation Energy (eV)	Oscillator Strength	Transition State
Adamantane	147	8.44	0.0034	HOMO(A)→LUMO(A) (48%), HOMO(B)→LUMO(B) (48%)
	147	8.44	0.0034	H-1(A)→LUMO(A) (48%), H-2(B)→LUMO(B) (48%)
C1 Substitution				
1-bora-adamantane	213	5.82	0.0034	H-1(A)→LUMO(A) (46%), HOMO(B)→LUMO(B) (46%)
	153	8.10	0.0952	H-4(A)→LUMO(A) (48%), H-4(B)→LUMO(B) (48%)
1,3-dibora-adamantane	235	5.28	0.0105	H-1(A)→LUMO(A) (48%), H-1(B)→LUMO(B) (48%)
	174	7.13	0.0269	H-6(A)→LUMO(A) (37%), H-3(A)→LUMO(A) (10%)
1,3,5-tribora-adamantane	258	4.81	0.0127	H-1(A)→LUMO(A) (49%), H-1(B)→LUMO(B) (49%)
	250	4.96	0.0101	H-3(A)→LUMO(A) (37%), H-2(B)→LUMO(B) (45%)
1,3,5,7-tetrabora-adamantane	423	2.93	0.0000	H-1(A)→LUMO(A) (48%), H-1(B)→LUMO(B) (48%)
	238	5.21	0.0006	H-5(A)→LUMO(A) (48%), H-5(B)→LUMO(B) (48%)
C2 Substitution				
2-bora-adamantane	184	6.74	0.0085	H-2→LUMO (98%)
	127	9.76	0.1442	H-3→L+1 (16%), H-1→L+2 (59%)
2,4-dibora-adamantane	267	4.64	0.0071	H-1(A)→LUMO(A) (46%), H-1(B)→LUMO(B) (46%)
	219	5.66	0.0137	H-2(A)→LUMO(A) (39%), H-2(B)→LUMO(B) (39%)
2,4,6-tribora-adamantane	293	4.23	0.0095	HOMO(A)→LUMO(A) (46%), HOMO(B)→LUMO(B) (46%)
	191	6.49	0.0171	H-5(A)→LUMO(A) (14%), H-2(A)→L+1(A) (21%)
2,4,6,8-tetrabora-adamantane	279	4.47	0.0084	H-1(A)→LUMO(A) (44%), H-1(B)→LUMO(B) (44%)
	206	6.02	0.0296	H-4(A)→LUMO(A) (30%), H-4(B)→LUMO(B) (30%)
2,4,6,8,9-pentabora-adamantane	252	4.92	0.0233	H-1(A)→LUMO(A) (33%), HOMO(A)→L+1(A) (14%)
	201	6.17	0.0501	H-4(A)→LUMO(A) (16%), H-3(A)→L+1(A) (22%)
2,4,6,8,9,10-hexabora-adamantane	252	4.92	0.0000	HOMO(A)→L+2(A) (41%), HOMO(B)→L+2(B) (41%)
	217	5.71	0.0331	H-1(A)→L+2(A) (48%), H-1(B)→L+2(B) (48%)

## Data Availability

All computational files are available on request to the corresponding author (S.L.M.).

## Supplementary Materials

The following supporting information can be downloaded at: https://www.mdpi.com/article/10.3390/molecules30091976/s1, Figure S1: Optimized structures of adamantane and B-substituted isomers of adamantane (B3LYP/6-31G(d)); Figure S2: Natural transition orbitals of some key isomers calculated using B3LYP/6-31G(d) (iso-surface value 0.02 e/Å); Table S1: Possible isomers for each combination of B-substituted adamantane showing structural configuration and therefore doping position (C1 or C2); Table S2: Geometric parameters (bond lengths in pm and bond angles in °) for boron-substituted adamantane series; Table S3: Electronic energies and energy differences (eV) for boron-substituted adamantane series calculated at B3LYP/6-31G(d) level; Table S4: Electronic energies and energy differences (eV) for the most critical transition of boron-substituted adamantane series calculated at CCSD/6-31G(d) level; Table S5: HOMO and LUMO orbitals energies (eV) for the for boron-substituted adamantane series; Table S6: Optical properties [wavelength (nm), excitation energy (eV) and oscillator strength] for boron-substituted adamantane series; Table S7: Optical properties [wavelength (nm), excitation energy (eV) and oscillator strength] for some boron-substituted adamantane isomers calculated at B3LYP/6-31+G(d,p); Table S8: Electronic energies (eV) of some key isomers calculated using different basis set with B3LYP functional.
