# Supplementary material for: Tuning the Energy Levels of Adamantane by Boron Substitution
Source: Molecules. 2025 Apr 29;30(9):1976. doi: 10.3390/molecules30091976 (PMC12073632; doi:10.3390/molecules30091976)
Supplement: Supplementary file 1 [file molecules-30-01976-s001.zip › molecules-3579017-supplementary.pdf]

Tuning the Energy Levels of Adamantane by Boron Substitution

Aminu H. Yusuf, Vladimir B. Golovko * and Sarah L. Masters *

Supplementary Information

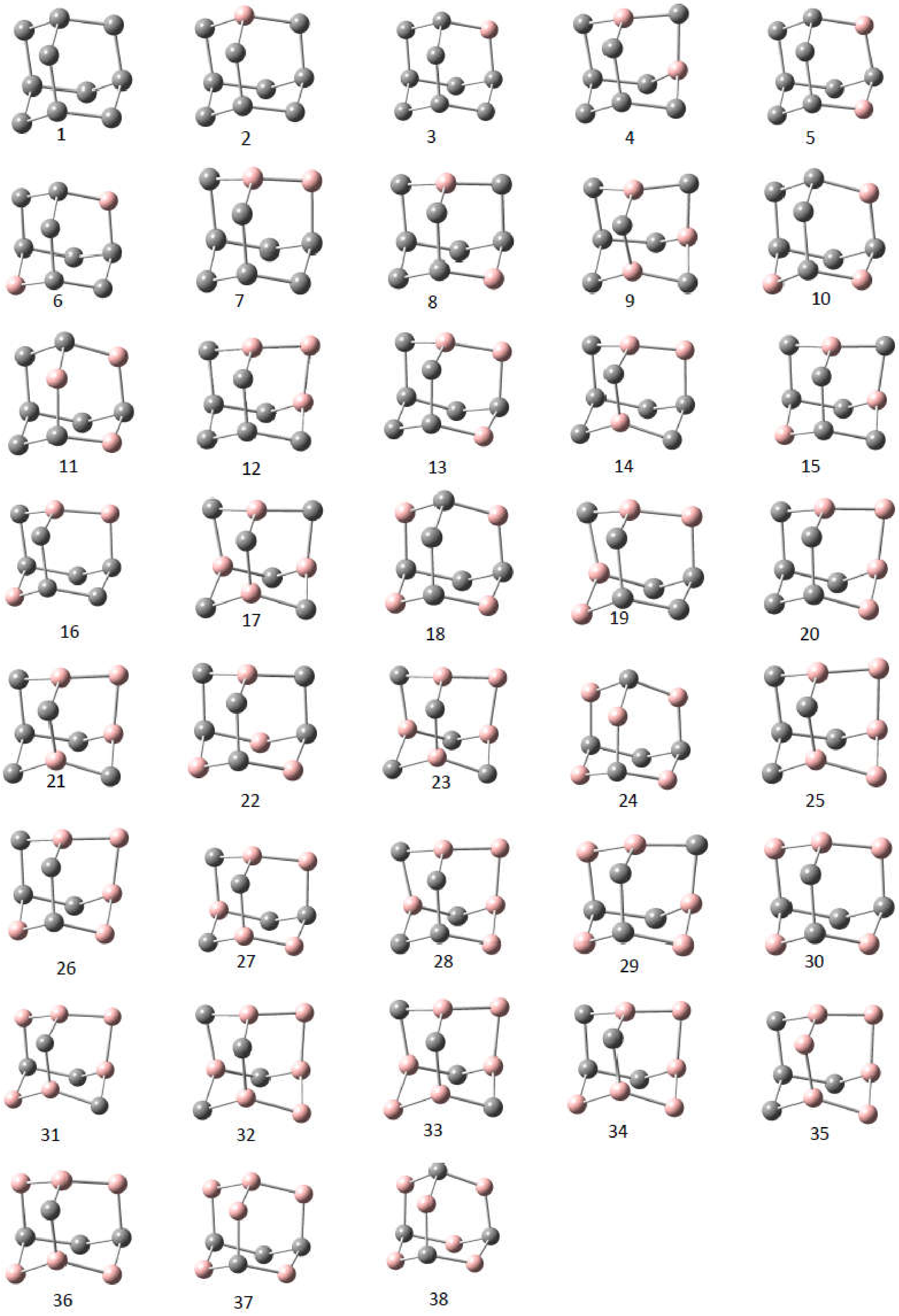

Figure S1: Optimized structures of adamantane and B-substituted isomers of adamantane (B3LYP/6-31G(d))

#	Molecules	Hole	particle
1	Pristine adamantane	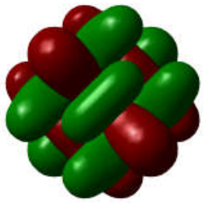	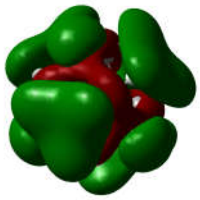
2	1-bora-adamantane	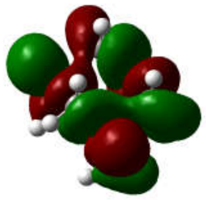	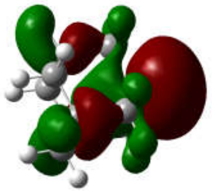
3	1,3-di-bora-adamantane	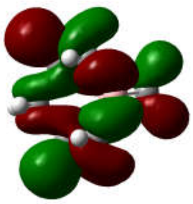	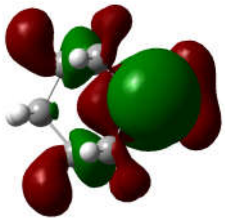
4	1,3,5-tri-bora-adamantane	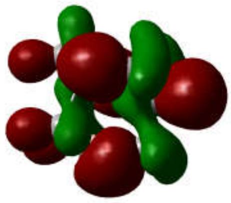	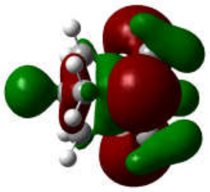
5	1,3,5,7-tetra-bora-adamantane	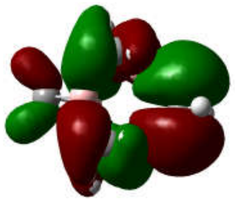	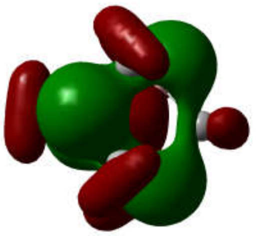

Figure S2: Natural transition orbitals of some key isomers calculated using B3LYP/6-31G(d). (Iso-surface value 0.02 e/Å)

Table S1: Possible isomers for each combination of B-substituted adamantane showing structural configuration and therefore doping position (C1 or C2).

#	# Boron	Molecular formula	Naming	Configuration of different atom types[a]
1	bare	$C_{10}H_{16}$	Adamantane	$(C1)_4(C2)_6$
2	B	$C_9H_{15}B$	1-bora-adamantane	$(C1)_3(B1)_1(C2)_6$
3		$C_9H_{15}B$	2-bora-adamantane	$(C1)_4(C2)_5(B2)_1$
4	2B	$C_8H_{14}B_2$	1,3-di-bora-adamantane	$(C1)_2(B1)_2(C2)_6$
5		$C_8H_{14}B_2$	2,4-di-bora-adamantane	$(C1)_4(C2)_4(B2)_2$
6		$C_8H_{14}B_2$	2,6-di-bora-adamantane	$(C1)_4(C2)_4(B2)_2$
7		$C_8H_{14}B_2$	1,2-di-bora-adamantane	$(C1)_3(B1)_1(C2)_5(B2)_1$
8		$C_8H_{14}B_2$	1,4-di-bora-adamantane	$(C1)_3(B1)_1(C2)_5(B2)_1$
9	3B	$C_7H_{13}B_3$	1,3,5-tri-bora-adamantane	$(C1)_1(B1)_3(C2)_6$
10		$C_7H_{13}B_3$	2,4,6-tri-bora-adamantane	$(C1)_4(C2)_3(B2)_3$
11		$C_7H_{13}B_3$	2,4,9-tri-bora-adamantane	$(C1)_2(B1)_2(C2)_5(B2)_1$
12		$C_7H_{13}B_3$	1,2,3-tri-bora-adamantane	$(C1)_2(B1)_2(C2)_5(B2)_1$
13		$C_7H_{13}B_3$	1,2,4-tri-bora-adamantane	$(C1)_2(B1)_1(C2)_4(B2)_2$
14		$C_7H_{13}B_3$	1,2,5-tri-bora-adamantane	$(C1)_2(B1)_2(C2)_5(B2)_1$
15		$C_7H_{13}B_3$	1,3,6-tri-bora-adamantane	$(C1)_2(B1)_2(C2)_5(B2)_1$
16		$C_7H_{13}B_3$	1,2,6-tri-bora-adamantane	$(C1)_3(B1)_1(C2)_4(B2)_2$
17	4B	$C_6H_{12}B_4$	1,3,5,7-tetra-bora-adamantane	$(B1)_4(C2)_6$
18		$C_6H_{12}B_4$	2,4,6,8-tetra-bora-adamantane	$(C1)_4(C2)_2(B2)_4$
19		$C_6H_{12}B_4$	1,2,6,7-tetra-bora-adamantane	$(C1)_2(B1)_2(C2)_4(B2)_2$
20		$C_6H_{12}B_4$	1,2,3,4-tetra-bora-adamantane	$(C1)_2(B1)_2(C2)_4(B2)_2$
21		$C_6H_{12}B_4$	1,2,3,5-tetra-bora-adamantane	$(C1)_1(B1)_3(C2)_5(B2)_1$
22		$C_6H_{12}B_4$	1,4,6,10-tetra-bora-adamantane	$(C1)_3(B1)_1(C2)_3(B2)_3$
23	5B	$C_5H_{11}B_5$	1,2,3,5,7-penta-bora-adamantane	$(B1)_4(C2)_5(B2)_1$
24		$C_5H_{11}B_5$	2,4,6,8,9-penta-bora-adamantne	$(C1)_4(C2)_1(B2)_5$
25		$C_5H_{11}B_5$	1,2,3,4,5-penta-bora-adamantane	$(C1)_1(B1)_3(C2)_4(B2)_2$
26		$C_5H_{11}B_5$	1,2,3,4,6-penta-bora-adamantane	$(C1)_2(B1)_2(C2)_3(B2)_3$
27		$C_5H_{11}B_5$	1,2,4,5,7-penta-bora-adamantane	$(C1)_1(B1)_3(C2)_4(B2)_2$
28		$C_5H_{11}B_5$	1,2,3,4,7-penta-bora-adamantane	$(C1)_1(B1)_3(C2)_4(B2)_2$
29		$C_5H_{11}B_5$	1,3,4,6,8-penta-bora-adamantane	$(C1)_2(B1)_2(C2)_3(B2)_3$
30		$C_5H_{11}B_5$	1,2,4,6,8-penta-bora-adamantane	$(C1)_3(B1)_1(C2)_2(B2)_4$
31	6B	$C_4H_{10}B_6$	1,2,3,5,6,7-hexa-bora-adamantane	$(B1)_4(C2)_4(B2)_2$
32		$C_4H_{10}B_6$	1,2,3,4,5,7-hexa-bora-adamantane	$(B1)_4(C2)_4(B2)_2$
33		$C_4H_{10}B_6$	1,2,3,5,6,8-hexa-bora-adamantane	$(C1)_1(B1)_3(C2)_3(B2)_3$
34		$C_4H_{10}B_6$	1,2,3,4,5,6-hexa-bora-adamantane	$(C1)_1(B1)_3(C2)_2(B2)_4$
35		$C_4H_{10}B_6$	1,2,3,4,5,9-hexa-bora-adamantane	$(C1)_1(B1)_3(C2)_3(B2)_3$
36		$C_4H_{10}B_6$	1,2,4,5,6,8-hexa-bora-adamantane	$(C1)_2(B1)_2(C2)_3(B2)_3$
37		$C_4H_{10}B_64$	1,2,4,6,8,9-hexa-bora-adamantane	$(C1)_3(B1)_1(C2)_1(B2)_5$
38		$C_4H_{10}B_6$	2,4,6,8,9,10-hexa-bora-adamantane	$(C1)_4(B2)_6$

[a] *Configuration indicates how many C1-type and C2-type carbons have been replaced with B1-type or B2-type. Hydrogen atoms have been omitted for clarity in the coding.*

Table S2: Geometric parameters (bond lengths in pm and bond angles in °) for boron-substituted adamantane series.

#	Molecules	Average bond length (pm)			Average bond angles (°)		
		C-H	C-C	C-B	C-C-C	H-C-H	C-B-C
1	Adamantane	108.8	153.7	-	109.7	106.8	-
2	1-bora-adamantane	108.7	154.1	157.7	109.4	108.3	116.2
3	2-bora-adamantane	108.5	155.1	157.6	110.7	106.4	114.1
4	1,3-di-bora-adamantane	108.6	156.8	158.2	110.1	106.7	117.0
5	2,4-di-bora-adamantane	108.9	154.5	158.2	108.7	106.3	113.8
6	2,6-di-bora-adamantane	108.7	155.1	158.2	110.2	105.8	112.1
7	1,2-di-bora-adamantane	108.7	155.2	158.6	109.4	107.8	115.1
8	1,4-di-bora-adamantane	108.9	156.3	158.7	109.6	107.5	116.1
9	1,3,5-tri-bora-adamantane	108.7	154.6	158.5	109.0	106.0	116.9
10	2,4,6-tri-bora-adamantane	108.7	154.5	158.3	112.1	105.6	114.1
11	2,4,9-tri-bora-adamantane	108.5	156.9	158.5	111.6	105.9	115.0
12	1,2,3-tri-bora-adamantane	109.7	157.3	162.1	111.4	107.9	114.0
13	1,2,4-tri-bora-adamantane	109.8	156.7	158.6	112.2	106.6	115.6
14	1,2,5-tri-bora-adamantane	109.7	157.5	158.9	110.8	108.2	117.1
15	1,3,6-tri-bora-adamantane	108.7	158	158.8	109.9	107.7	116.2
16	1,2,6-tri-bora-adamantane	108.7	157.1	158.7	109.5	106.8	117.9
17	1,3,5,7-tetra-bora-adamantane	108.5	-	159.8	-	110.6	116.7
18	2,4,6,8-tetra-bora-adamantane	108.6	157.1	158.3	113.8	104.9	114.7
19	1,2,6,7-tetra-bora-adamantane	109.7	158.2	159.1	110.5	105.8	117.1
20	1,2,3,4-tetra-bora-adamantane	109.8	156.5	159.8	111.3	107.6	114.2
21	1,2,3,5-tetra-bora-adamantane	108.7	156.9	159.7	110.7	108.4	115.4
22	1,4,6,10-tetra-bora-adamantane	108.7	159.2	158.8	-	107.5	116.8
23	1,2,3,5,7-penta-bora-adamantane	108.7	-	160.5	-	110.2	116.7
24	2,4,6,8,9-penta-bora-adamantne	109.5	156.7	157.5	111.3	105.6	117.7
25	1,2,3,4,5-penta-bora-adamantane	109.6	156.9	159.9	111.2	108.4	114.8
26	1,2,3,4,6-penta-bora-adamantane	109.8	158.7	159.6	110.6	107.2	115.9
27	1,2,4,5,7-penta-bora-adamantane	109.6	154.6	157.8	-	108.3	115.4
28	1,2,3,4,7-penta-bora-adamantane	108.8	157.7	160.6	110.5	108.9	116.7
29	1,3,4,6,8-penta-bora-adamantane	108.7	159.9	159.9	-	108	117.6
30	1,2,4,6,8-penta-bora-adamantane	108.6	158.3	159.7	115.8	105.7	116.4
31	1,2,3,5,6,7-hexa-bora-adamantane	108.8	-	160.5	-	109.6	116.4
32	1,2,3,4,5,7-hexa-bora-adamantane	109.7	-	159.6	-	110.5	116.5
33	1,2,3,5,6,8-hexa-bora-adamantane	108.7	160	160.5	-	108.8	116.9
34	1,2,3,4,5,6-hexa-bora-adamantane	109.5	158.3	162.8	110.7	108.1	115.5
35	1,2,3,4,5,9-hexa-bora-adamantane	109.6	156.4	161.7	111.6	107.3	-
36	1,2,4,5,6,8-hexa-bora-adamantane	108.7	157.9	160.9	117.6	107.3	-
37	1,2,4,6,8,9-hexa-bora-adamantane	108.7	156.6	159.0	114.0	105.2	118.7
38	2,4,6,8,9,10-hexa-bora-adamantane	108.2	-	158.1	-	-	118.6

Classification: In-Confidence

Table S3: Electronic energies and energy differences (eV) for boron-substituted adamantane series calculated at B3LYP/6-31G(d) level.

#	Molecule	E_0	E_+	E_-	ΔE_1	ΔE_2
1	Adamantane	-10632.18	-10623.09	-10628.82	9.10	-3.36
2	1-bora-adamantane	-10254.77	-10246.20	-10253.60	8.57	-1.17
3	2-bora-adamantane	-10254.56	-10246.02	-10253.45	8.54	-1.11
4	1,3-di-bora-adamantane	-9877.72	-9868.85	-9877.03	8.87	-0.69
5	2,4-di-bora-adamantane	-9877.23	-9868.22	-9876.98	9.02	-0.25
6	2,6-di-bora-adamantane	-9876.92	-9868.13	-9875.93	8.79	-0.99
7	1,2-di-bora-adamantane	-9876.67	-9868.25	-9875.37	8.43	-1.30
8	1,4-di-bora-adamantane	-9877.13	-9868.69	-9876.38	8.44	-0.74
9	1,3,5-tri-bora-adamantane	-9500.93	-9492.21	-9501.44	8.72	0.51
10	2,4,6-tri-bora-adamantane	-9499.90	-9491.19	-9500.50	8.71	0.60
11	2,4,9-tri-bora-adamantane	-9500.19	-9491.66	-9501.15	8.53	0.96
12	1,2,3-tri-bora-adamantane	-9499.23	-9491.26	-9499.20	7.97	-0.02
13	1,2,4-tri-bora-adamantane	-9499.30	-9491.39	-9499.54	7.91	0.24
14	1,2,5-tri-bora-adamantane	-9499.60	-9491.62	-9499.47	7.98	-0.13
15	1,3,6-tri-bora-adamantane	-9500.10	-9494.31	-9499.87	5.80	-0.23
16	1,2,6-tri-bora-adamantane	-9499.77	-9491.26	-9500.13	8.51	0.36
17	1,3,5,7-tetra-bora-adamantane	-9124.18	-9115.40	-9125.44	8.78	1.27
18	2,4,6,8-tetra-bora-adamantane	-9122.80	-9114.07	-9123.36	8.73	0.56
19	1,2,6,7-tetra-bora-adamantane	-9121.46	-9113.50	-9121.46	7.97	-0.004
20	1,2,3,4-tetra-bora-adamantane	-9121.33	-9113.39	-9121.61	7.94	0.29
21	1,2,3,5-tetra-bora-adamantane	-9122.34	-9114.45	-9123.03	7.89	0.69
22	1,4,6,10-tetra-bora-adamantane	-9122.63	-9114.15	-9123.84	8.48	1.21
23	1,2,3,5,7-penta-bora-adamantane	-8745.59	-8737.73	-8746.89	7.86	1.30
24	2,4,6,8,9-penta-bora-adamantne	-8746.72	-8737.79	-8747.82	8.93	0.14
25	1,2,3,4,5-penta-bora-adamantane	-8743.94	-8736.31	-8744.80	7.63	0.86
26	1,2,3,4,6-penta-bora-adamantane	-8743.94	-8735.88	-8744.60	8.06	0.66
27	1,2,4,5,7-penta-bora-adamantane	-8744.56	-8736.55	-8745.93	8.01	1.37
28	1,2,3,4,7-penta-bora-adamantane	-8744.41	-8736.46	-8745.32	7.96	0.91
29	1,3,4,6,8-penta-bora-adamantane	-8744.32	-8736.18	-8744.94	8.14	0.62
30	1,2,4,6,8-penta-bora-adamantane	-8744.29	-8736.33	-8744.87	7.96	0.58
31	1,2,3,5,6,7-hexa-bora-adamantane	-8367.04	-8358.92	-8368.35	8.12	1.31
32	1,2,3,4,5,7-hexa-bora-adamantane	-8367.16	-8359.49	-8368.51	7.66	1.35
33	1,2,3,5,6,8-hexa-bora-adamantane	-8366.50	-8358.45	-8367.53	8.05	1.03
34	1,2,3,4,5,6-hexa-bora-adamantane	-8366.00	-8358.17	-8367.03	7.83	1.03
35	1,2,3,4,5,9-hexa-bora-adamantane	-8365.66	-8358.37	-8366.70	7.30	1.03
36	1,2,4,5,6,8-hexa-bora-adamantane	-8366.08	-8358.11	-8367.04	7.97	0.97
37	1,2,4,6,8,9-hexa-bora-adamantane	-8367.64	-8359.10	-8368.83	8.53	1.19
38	2,4,6,8,9,10-hexa-bora-adamantane	-8370.50	-8361.30	-8371.43	9.20	0.93

Table S4: Electronic energies and energy differences (eV) for the most critical transition of boron-substituted adamantane series calculated at CCSD/6-31G(d) level.

#	Molecule	DFT Energy (B3LYP)	CCSD(T) Energy	Δ (DFT–CCSD(T))	% Error (Relative to CCSD(T))
	Pristine Adamantane	−10632.18	−10601.81	−30.38	0.29%
1	1,3-di-bora-adamantane	−9877.73	−9852.07	−25.66	0.26%
2	1,3,5-tri-bora-adamantane	−9500.93	−9475.77	−25.16	0.27%
3	2,4-di-bora-adamantane	−9877.23	−9848.1	−29.13	0.30%
4	2,4,6-tri-bora-adamantane	−9499.9	−9471.33	−28.57	0.30%

*Notes: % Error = [(DFT−CCSD(T))/CCSD(T)]×100 for absolute energies.

Table S5: HOMO and LUMO orbitals energies (eV) for the boron-substituted adamantane series.

#	Molecule	HOMO	LUMO	HOMO-LUMO Gap
1	Adamantane	-7.44	1.88	9.32
2	1-bora-adamantane	-7.05	-0.09	6.96
3	2-bora-adamantane	-7.09	-0.62	6.47
4	1,3-di-bora-adamantane	-6.95	-.1.16	5.79
5	2,4-di-bora-adamantane	-7.42	-1.89	5.53
6	2,6-di-bora-adamantane	-7.11	-0.70	6.41
7	1,2-di-bora-adamantane	-6.61	-1.03	5.58
8	1,4-di-bora-adamantane	-6.97	-0.85	6.12
9	1,3,5-tri-bora-adamantane	-7.03	-2.05	4.98
10	2,4,6-tri-bora-adamantane	-7.09	-1.86	5.23
11	2,4,9-tri-bora-adamantane	-7.28	-2.21	5.07
12	1,2,3-tri-bora-adamantane	-6.25	-1.36	4.89
13	1,2,4-tri-bora-adamantane	-6.56	-1.57	4.99
14	1,2,5-tri-bora-adamantane	-6.50	-1.39	5.11
15	1,3,6-tri-bora-adamantane	-7.56	-1.25	6.31
16	1,2,6-tri-bora-adamantane	-6.94	-2.72	4.22
17	1,3,5,7-tetra-bora-adamantane	-7.13	-3.03	4.10
18	2,4,6,8-tetra-bora-adamantane	-7.14	-1.96	5.18
19	1,2,6,7-tetra-bora-adamantane	-6.37	-1.55	4.82
20	1,2,3,4-tetra-bora-adamantane	-6.28	-1.56	4.72
21	1,2,3,5-tetra-bora-adamantane	-6.19	-2.21	3.98
22	1,4,6,10-tetra-bora-adamantane	-7.13	-2.63	4.50
23	1,2,3,5,7-penta-bora-adamantane	-6.18	-3.05	3.13
24	2,4,6,8,9-penta-bora-adamantne	-7.36	-1.61	5.75
25	1,2,3,4,5-penta-bora-adamantane	-5.97	-2.39	3.58
26	1,2,3,4,6-penta-bora-adamantane	-6.44	-1.92	4.50
27	1,2,4,5,7-penta-bora-adamantane	-6.64	-2.65	3.99
28	1,2,3,4,7-penta-bora-adamantane	-6.3	-2.32	3.98
29	1,3,4,6,8-penta-bora-adamantane	-6.58	-1.97	4.61
30	1,2,4,6,8-penta-bora-adamantane	-6.44	-2.14	4.30
31	1,2,3,5,6,7-hexa-bora-adamantane	-6.35	-3.05	3.30
32	1,2,3,4,5,7-hexa-bora-adamantane	-6.03	-3.09	2.94
33	1,2,3,5,6,8-hexa-bora-adamantane	-6.42	-2.43	3.99
34	1,2,3,4,5,6-hexa-bora-adamantane	-6.17	-2.50	3.67
35	1,2,3,4,5,9-hexa-bora-adamantane	-5.70	-2.60	3.10
36	1,2,4,5,6,8-hexa-bora-adamantane	-6.39	-2.54	3.85
37	1,2,4,6,8,9-hexa-bora-adamantane	-7.2	-1.83	5.37
38	2,4,6,8,9,10-hexa-bora-adamantane	-7.71	-1.40	6.31

Classification: In-Confidence

Table S6: Optical properties [wavelength (nm), excitation energy (eV) and oscillator strength] for boron-substituted adamantane series.

#	Molecules	Absorption Wavelength (nm)	Excitation Energy (eV)	Oscillator Strength	Major Excitation
1	Adamantane	165	7.52	0.0046	HOMO→LUMO (88%)
2	1-bora-adamantane	222	5.59	0.0031	HOMO→LUMO (97%)
3	2-bora-adamantane	245	5.06	0.0013	HOMO→LUMO (98%)
4	1,3-di-bora-adamantane	248	5.00	0.0102	HOMO-1→LUMO (98%)
5	2,4-di-bora-adamantane	279	4.44	0.0075	HOMO-1→LUMO (98%)
6	2,6-di-bora-adamantane	233	5.32	0.0109	HOMO→LUMO (93%)
7	1,2-di-bora-adamantane	294	4.22	0.0059	HOMO→LUMO (98%)
8	1,4-di-bora-adamantane	243	510	0.0033	HOMO-1→LUMO (96%)
9	1,3,5-tri-bora-adamantane	279	4.44	0.0114	HOMO-1→LUMO (99%)
10	2,4,6-tri-bora-adamantane	298	416	0.0105	HOMO→LUMO (98%)
11	2,4,9-tri-bora-adamantane	308	4.03	0.0073	HOMO→LUMO (99%)
12	1,2,3-tri-bora-adamantane	314	3.95	0.0254	HOMO→LUMO (98%)
13	1,2,4-tri-bora-adamantane	259	4.78	0.0061	HOMO→LUMO+1 (87%)
14	1,2,5-tri-bora-adamantane	277	4.47	0.0056	HOMO→LUMO+1 (94%)
15	1,3,6-tri-bora-adamantane	240	5.17	0.0059	HOMO-1→LUMO (89%)
16	1,2,6-tri-bora-adamantane	392	3.16	0.0021	HOMO→LUMO (100%)
17	1,3,5,7-tetra-bora-adamantane	420	2.95	0.0000	HOMO→LUMO (100%)
18	2,4,6,8-tetra-bora-adamantane	297	4.18	0.0109	HOMO→LUMO (98%)
19	1,2,6,7-tetra-bora-adamantane	366	3.39	0.0004	HOMO→LUMO (100%)
20	1,2,3,4-tetra-bora-adamantane	340	3.68	0.0139	HOMO→LUMO (93%)
21	1,2,3,5-tetra-bora-adamantane	541	2.29	0.0037	HOMO→LUMO (98%)
22	1,4,6,10-tetra-bora-adamantane	603	2.06	0.0001	HOMO→LUMO (100%)
23	1,2,3,5,7-penta-bora-adamantane	607	2.04	0.0015	HOMO→LUMO (100%)
24	2,4,6,8,9-penta-bora-adamantne	264	4.70	0.0002	HOMO→LUMO+1 (81%)
25	1,2,3,4,5-penta-bora-adamantane	340	2.39	0.0076	HOMO-1→LUMO (97%)
26	1,2,3,4,6-penta-bora-adamantane	324	3.82	0.0062	HOMO→LUMO+1 (89%)
27	1,2,4,5,7-penta-bora-adamantane	420	2.95	0.0015	HOMO→LUMO (98%)
28	1,2,3,4,7-penta-bora-adamantane	631	1.97	0.0021	HOMO→LUMO (100%)
29	1,3,4,6,8-penta-bora-adamantane	511	2.43	0.0002	HOMO→LUMO (100%)
30	1,2,4,6,8-penta-bora-adamantane	449	2.76	0.0047	HOMO→LUMO (100%)
31	1,2,3,5,6,7-hexa-bora-adamantane	557	2.23	0.0022	HOMO→LUMO (100%)
32	1,2,3,4,5,7-hexa-bora-adamantane	399	3.10	0.0028	HOMO→LUMO+1 (91%)
33	1,2,3,5,6,8-hexa-bora-adamantane	813	1.53	0.0015	HOMO→LUMO (100%)
34	1,2,3,4,5,6-hexa-bora-adamantane	495	2.51	0.0037	HOMO→LUMO (98%)
35	1,2,3,4,5,9-hexa-bora-adamantane	315	3.93	0.0057	HOMO-1→LUMO (46%)
36	1,2,4,5,6,8-hexa-bora-adamantane	519	2.39	0.0079	HOMO→LUMO (98%)
37	1,2,4,6,8,9-hexa-bora-adamantane	755	1.64	0.0048	HOMO→LUMO (98%)
38	2,4,6,8,9,10-hexa-bora-adamantane	243	5.10	0.0000	HOMO→LUMO (60%)

Table S7: Optical properties [wavelength (nm), excitation energy (eV) and oscillator strength] for some boron-substituted adamantane isomers calculated at B3LYP/6-31+G(d,p).

#	Molecules	Absorption Wavelength (nm)	Excitation Energy (eV)	Oscillator Strength	Major Excitation
1	Pristine adamantane	187	6.63	0.0049	HOMO→LUMO (87%)
2	1-bora-adamantane	197	6.29	0.0076	HOMO→LUMO (99%)
3	1,3-di-bora-adamantane	250	4.96	0.0094	HOMO→LUMO (100%)
4	1,3,5-tri-bora-adamantane	273	4.56	0.0102	HOMO-1→LUMO (94%)
5	1,3,5,7-tetra-bora-adamantane	504	2.46	0.0000	HOMO-1→LUMO (100)%)

Table S8: Electronic energies (eV) of some key isomers calculated using different basis set with B3LYP functional.

#	Molecule	6-31G(d)	6-31G(d)-gd3	6-31+G(d)	6-311+G(d)
	Pristine adamantane	-10632.18	-10632.91	-10632.78	-10,634.66
1	1-bora-adamantane	-10254.77	-10255.46	-10.255.58	-10257.39
2	1,3-di-bora-adamantane	-9877.72	-9878.37	-9878.55	-9880.29
3	1,3,5-tri-bora-adamantane	-9500.93	-9501.53	-9501.77	-9503.42
4	1,3,5,7-tetra-bora-adamantane	-9124.18	-97124.73	-9125.03	-9126.56

Classification: In-Confidence
